# Efficacy and safety of isosorbide mononitrate plus misoprostol compared to misoprostol alone in the management of the first and second trimester abortion: a systematic review and meta-analysis

**DOI:** 10.1186/s12884-024-06614-9

**Published:** 2024-06-10

**Authors:** Somayeh Makvandi, Leila Karimi, Masoumeh Safyari, Mona Larki

**Affiliations:** 1 Department of Midwifery, Menopause Andropause Research Center, Ahvaz Jundishapur University of Medical Sciences, Ahvaz, Iran; 2 Behavioral Sciences Research Center, Life Style Institute, Faculty of Nursing, Baqiyatallah University of Medical Sciences, Tehran, Iran; 3 Department of Women’s Health Nursing and Midwifery, Graduate School of Medicine, Tohoku University, Sendai, Japan; 4 Nursing and Midwifery Care Research Center, Mashhad University of Medical Sciences, Mashhad, Iran; 5 Department of Midwifery, School of Nursing and Midwifery, Mashhad University of Medical Sciences, Mashhad, Iran

**Keywords:** Isosorbide Mononitrate, Misoprostol, Abortion, Pregnancy

## Abstract

**Background:**

However, misoprostol is often used to terminate a pregnancy, but it can also cause side effects. Isosorbide mononitrate (ISMN) can help the cervix mature by increasing the production of prostaglandin E2 and vasodilation. Considering that the results of studies in this field are contradictory, it is the purpose of this study to evaluate the efficacy and safety of vaginal ISMN plus misoprostol compared to misoprostol alone in the management of first- and second-trimester abortions.

**Method:**

The search process was conducted for MEDLINE through the PubMed interface, Scopus, Web-of-Science, Science Direct, the Cochrane Central Register of Controlled Trials (CENTRAL), Google Scholar, ClinicalTrials.gov, and the World Health Organization International Clinical Trials Registry Platform until November 10, 2023. Our assessment of bias was based on version 2 of the risk-of-bias tool (RoB2) for randomized trials and our level of evidence quality was determined by GRADE. Meta-analysis of all data was carried out using Review Manager (RevMan) version 5.1.

**Result:**

Seven randomized clinical trials were included in the systematic review and three in the meta-analysis, with mixed quality. The results of the meta-analysis revealed that in the second-trimester abortion, the inclusion of ISMN in conjunction with vaginal misoprostol results in a noteworthy reduction in the induction abortion interval, specifically by 4.21 h (95% CI: -7.45 to -0.97, *P* = 0.01). The addition of vaginal ISMN to misoprostol, compared to vaginal misoprostol alone, increased the odds of a completed abortion by 3.76 times. (95% CI: 1.08 to 13.15, *P* = 0.04).

**Conclusion:**

The findings of this study can offer valuable insights aimed at enhancing counseling and support for non-surgical methods of medication abortion within professional settings. Moreover, it improves the effectiveness of clinical treatment and reduces the occurrence of unnecessary surgical interventions in the abortion management protocol.

## Introduction

Miscarriage is a prevalent challenge experienced by women during pregnancy and stands as one of the primary contributors to maternal mortality [1]. Approximately 15% of all clinically diagnosed pregnancies fall into this category [2]. In 2020, 51% of legal abortions registered with the Centers for Disease Control and Prevention were early-term abortions [3]. Medical abortion treatment is effective in women with missed abortions or empty sacs [4, 5]. However, misoprostol is often used to terminate a pregnancy in the second trimester [6], but it can also cause side effects like vomiting, nausea, and vaginal bleeding [7–9]. Many other substances have been utilized in the management of abortion, including oxytocin, corticosteroids, estrogens, and relaxin [10] whereas nitric oxide donors (NO donors) are relatively new short-lived free radical gas compounds [11].

The isosorbide mononitrate (ISMN) a NO donor, facilitates cervical dilation by promoting the secretion of prostaglandin E2 and dilating the blood vessels in that area. When used carefully, it is safe, does not stimulate the myometrium, and causes few life-threatening side effects [12].

Previous clinical trials have confirmed that the combination of ISMN and misoprostol administration is associated with a higher chance of success for second-trimester abortions when compared to misoprostol administration alone [10, 13–17]. However, a study from the UK in 2001 found that using misoprostol with nitric oxide donor was not better than using misoprostol alone for cervical maturation in the first trimester [18]. The results of other studies also showed that the vaginal use of ISMN before the vaginal administration of misoprostol does not significantly increase the number of pregnancy terminations in the second trimester [19, 20].

Given the controversy regarding the matter, the existence of studies with small sample sizes, and the significance of incorporating high-quality recommendations on this topic to draw clear conclusions to estimate the efficacy and safety of ISMN plus misoprostol compared to misoprostol alone in the management of first- and second-trimester abortions.

## Method

This systematic review and meta-analysis adhere to the 2020 Preferred Reporting Items for Systematic Review and Meta-Analyses (PRISMA) standards [21]. Furthermore, we adhered to the procedures outlined in the Cochrane Handbook for Systematic Reviews of Interventions [22].

### Search strategy

For original publications about " efficacy and safety of combined misoprostol and ISMN versus only misoprostol for cervical ripening in both the first and second-trimester abortion,” a search was conducted until November 10, 2023. The search process was conducted for MEDLINE through the PubMed interface, Scopus, Web-of-Science, Science Direct, the Cochrane Central Register of Controlled Trials (CENTRAL), and Google Scholar. To identify additional ongoing or completed trials, we conducted searches on ClinicalTrials.gov and the World Health Organization International Clinical Trials Registry Platform (apps.who.int/trial search/), which encompasses various trial registers, including ISRCTN and ClinicalTrials.gov.The search terms encompassed MESH, entry phrases, and keyword choices made by specialists. They comprised: Misoprostol, Cytotec, Isosorbide Mononitrate, Nitric oxide donor, first trimester, second trimester, abortion, missed abortion, miscarriage, termination of pregnancy, pregnancy loss, surgical evacuation, curettage, anembryonic, cervical ripening, termination of pregnancy, and second-trimester termination.

### Inclusion and exclusion criteria

Articles were included that met the following criteria: (a) Type of study: randomized clinical trials (RCTs); (b) Participants: pregnant women candidates for the first and second-trimester abortion without vaginal bleeding, uterine contractions, cervical dilatation, and any clinical symptoms that indicate the beginning of the abortion process; (c) Type of intervention: administration of vaginal ISMN at any dose and length of time plus with vaginal misoprostol (intervention group) compared with vaginal misoprostol alone (control group); (C) Outcomes: induction abortion interval, completed abortion rate (primary outcomes), and side effects of the ISMN and misoprostol compared to misoprostol (secondary outcome).

A successful abortion refers to the complete expulsion of all pregnancy-related products without the need for surgical intervention.

Exclusion criteria included (a) studies conducted on animals; (b) lack of access to full text; (C) letters to the editor; commentary; articles presented at conferences; preprint articles; and retracted articles. There were no limitations for setting, language, or time.

### Data abstraction

Two independent investigators reviewed the main output of the search process in terms of title and abstract after removing duplicate articles and rejecting unrelated items. The complete texts of the remaining articles were subsequently reviewed. Only full texts that satisfied the criteria for eligibility remained after irrelevant ones were removed. In cases where there was disagreement between reviewers, the variations were discussed between the two appraisers to get a final, unified opinion. If disagreement persisted, a third person would join the debate.

### Data extraction

The research team initially constructed a data extraction tool, and the data was extracted based on the items. This was done to extract the data from the articles in an integrated manner. The first author’s name, the publication year, the country, the type of study, the sample size, the sample characteristics, the intervention, the comparison, the tools used to collect the data, the quality assessment, and the outcomes were all listed. Two independent researchers (LK and ML) used independent pairwise ratings to perform this assessment. Disagreements were resolved through further discussion and, when resolution was not achievable through dialogue, consultation with an independent third coder was sought.

### Risk of bias assessment

Two authors autonomously evaluated the quality of the encompassed studies. Two authors autonomously assessed the quality of the incorporated studies. The risk of bias for RCTs was evaluated using Version 2 of the risk-of-bias tool (RoB2) for randomized trials as outlined in the Cochrane Handbook [23].

The ROB2 comprises five domains that encompass potential sources of bias in study outcomes, namely [1] the randomization process [2], deviations from intended interventions [3], missing outcome data [4], measurement of the outcome, and [5] selection of the reported result. Each domain, as well as the overall assessment of each study, is categorized as exhibiting either a low risk of bias, some concerns regarding the risk of bias, or a high risk of bias.

### Grading of evidence

According to the Grading of Recommendations Assessment, Development, and Evaluation (GRADE) criteria [24], two authors assessed the level of evidence for the outcomes to judge the reliability of the results. For randomized trials, the assessment was divided into five panels: risk of bias, inconsistency, indirectness, imprecision, and publication bias. The level of evidence quality was classified into four grades: high, moderate, low, and very low.

### Statistical analyses

Meta-analysis of all data was carried outperformed using Review Manager (RevMan) version 5.1. For the same outcome that had a mean and standard deviation, if the same assessment scale was used between studies, the mean difference (MD) was utilized to estimate the effect size, with 95% confidence intervals (CI) to express the confidence level. We used the odds ratio (OR) with a 95% CI to express dichotomous data. Heterogeneity between studies was evaluated utilizing Chi-squared and I-squared, and I-squared > 50% was considered to be significantly heterogeneous. Where no significant statistical heterogeneity was identified, the fixed effects estimate was used preferentially as the summary measure. The forest plots served as a means to succinctly present information from individual studies, visually indicate the degree of study heterogeneity, and depict the estimated common effect, amalgamating these elements into a single figure. Publication bias was not evaluated given the limited number of studies incorporated in each forest plot. Moreover, subgroup analyses were set up to explore whether the results of the effect values were the same under different conditions, and sensitivity analysis was used to verify the reliability of the meta-analysis results and reduce heterogeneity.

### Ethical considerations

We rigorously adhered to all research ethics requirements in the current study. The authors exerted attention to prevent plagiarism and refrain from manipulating the data for personal gain. The research team thoroughly addressed all ethical concerns throughout the process of identifying, screening, extracting, and analyzing the data.

## Result

### Search results

**Fig. 1:**
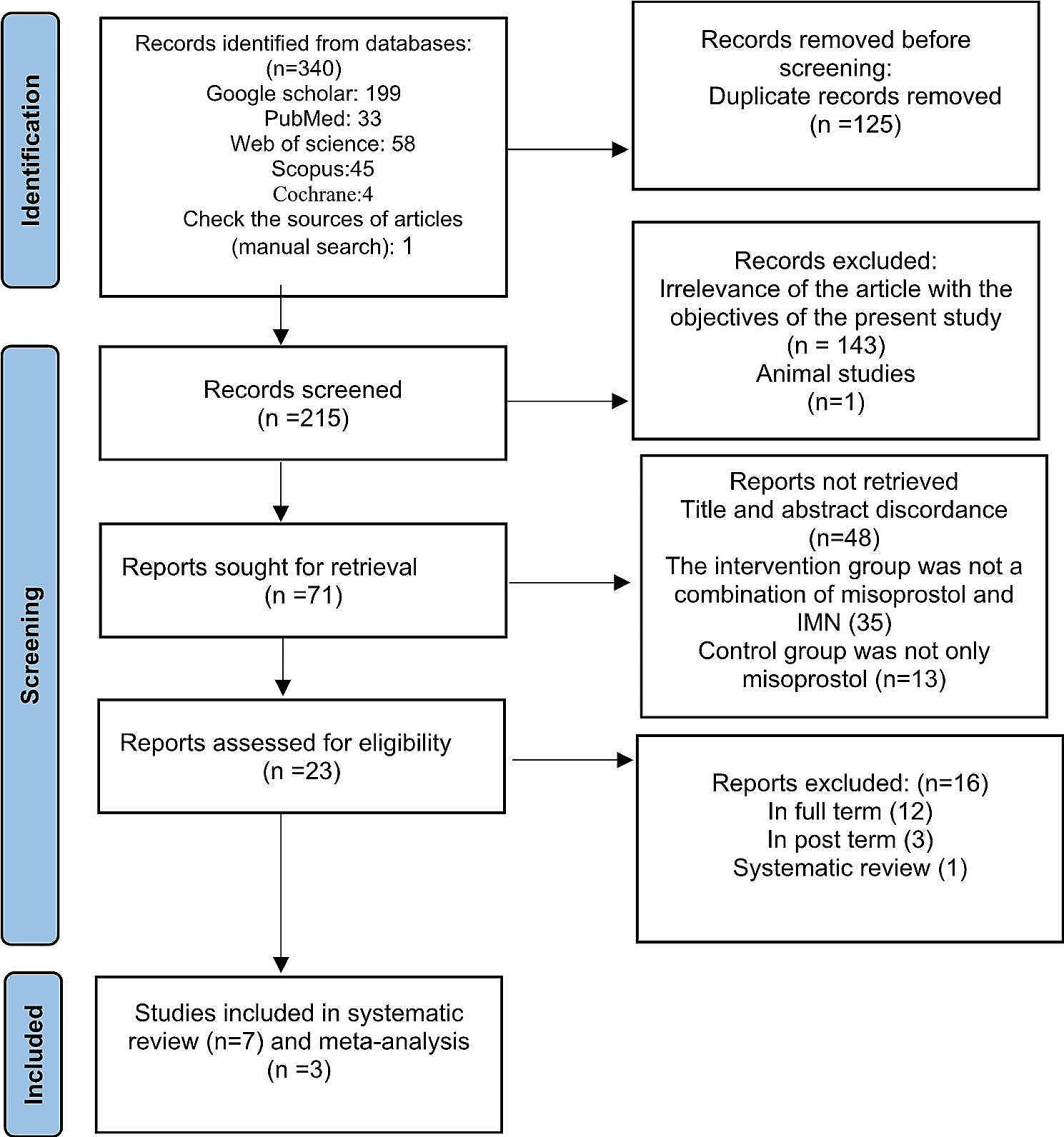
Flowchart of the process of selecting articles based on PRISMA 2020

Electronic databases yielded 340 articles in the initial search. After removing duplicates, 215 articles remained. Figure 1 provides a detailed explanation of the stages involved in the selection of articles. After a full-text review, 7 studies were included in the systematic review and 3 in the meta-analysis (Fig. 1).

### Study characteristics

The characteristics of the included studies are shown in Table 1. All seven included studies were randomized controlled trials. The studies were conducted in various countries, including India [16, 17], Egypt [13, 14, 25], the UK [18], and Greece [15]. The studies were published from 2001 [18] to 2023 [13]. The sample sizes ranged from 54 [13] to 160 [14] per study. The interventions included the combination of vaginal ISMN and misoprostol versus misoprostol alone for first and second-trimester abortions.

**Table 1:** Characteristics of included studies

First author (year)	Country	Subjects	Trimester of pregnancy	Intervention group	Comparison(s)	Findings
Abdel Dayem (2023) [13]	Egypt	Women with missed abortions and the absence of uterine activity, vaginal bleeding, cervical dilatation, and effacement	First trimester	*N* = 27 200 mcg Misoprostol plus 20 mg ISMN in the posterior vaginal fornix (single dose) 4 h before the surgical evacuation	*N* = 27 400 mcg Misoprostol in the posterior vaginal fornix 4 h before the surgical evacuation	Both groups were effective in achieving cervical ripening in terms of effacement, dilatation, and softening but the combination group was more effective than the Misoprostol group. The operative duration was statistically significantly shorter in the combination group than in the Misoprostol group. Abdominal pain was statistically significant and more prominent in group Misoprostol group while headache was more significant in the combination therapy group
Khalifa (2021) [25]	Egypt	Women scheduled for termination of anembryonic, maternal age ≥ 20 years, no vaginal bleeding, no dilation of the internal os, mean gestational sac diameter greater than 25 mm, and no yolk sac	First trimester	*N* = 54 800 mcg Misoprostol plus 40 mg ISMN tablets in the posterior vaginal fornix (single dose)	*N* = 54 800 mcg Misoprostol plus placebo were applied in the posterior vaginal fornix	A combination of Misoprostol and ISMN is more effective than misoprostol alone in the termination of anembryonic pregnancy and with less frequency of side effects.
Ledingham (2001) [18]	United Kingdom	Primigravid women undergoing surgical termination by vacuum aspiration	First trimester	*N* = 22 400 mg Misoprostol plus 40 mg ISMN intravaginally (single dose)	*N* = 21 400 mcg Misoprostol intravaginally	No discernible benefits were observed in combining misoprostol with ISMN as opposed to using misoprostol alone for pre-operative cervical ripening in the first trimester. There was no difference in the incidence of headaches between the ISMN and combination groups. Women who received combination therapy experienced the side effects of each agent used alone
					*N* = 22 40 mg ISMN intravaginally	
Atalla (2022) [14]	Egypt	18–35 years old women with missed abortion or IUFD confirmed by ultrasound, singleton pregnancy, unscarred uterus, normal uterus and cervix on clinical examination, uterine cervix is not dilated, no cervical dilatation or vaginal bleeding	Second trimester	*N* = 80 400 mcg Misoprostol plus 20 mg ISMN vaginally; Then one 200 mcg Misoprostol every 4–6 h to a maximum of four doses or until reaching cervical ripening.	*N* = 80 400 mcg Misoprostol vaginally then one 200 mcg Misoprostol every 4–6 h to a maximum of 4 doses or until reaching cervical ripening.	Combination therapy gives better results regarding cervical consistency improvement, cervical dilatation, effacement, the whole induction time, and the number of misoprostol doses needed to complete expulsion when compared to misoprostol alone Fewer side effects such as abdominal pain. among the misoprostol plus ISMN group compared to the Misoprostol group Headache showed a significant difference with a higher proportion among the Misoprostol plus ISMN group compared to the Misoprostol group
Mousiolis (2013) [15]	Greece	Women with elective terminations of pregnancy due to a major fetal anomaly	Second trimester	*N* = 30 200 mcg Misoprostol plus 60 mg ISMN vaginally., and if the abortion was not completed, another 60 mg was administered, 6 h after the first dose	*N* = 30 400 mcg Misoprostol per vaginum and 400 mcg misoprostol per os, followed by 6 h of administration of 400 mcg Misoprostol per vaginum until the fetus was expelled	The mean duration from induction to complete abortion interval was comparatively shorter in the group subjected to combination therapy.
Rukkayal Fathima (2016) [17]	India	100 pregnant women undergoing induced abortion.	Second trimester	*N* = 50 400 mcg of Misoprostol plus 40 mg of ISMN intravaginally. Repeat doses consist of a combination of 400 mcg of misoprostol and 20 mg ISMN every 4 h for an upper limit of 4 doses	*N* = 50 400 mcg of Misoprostol intravaginally every 4 h for maximum 4 doses	The mean duration between induction and abortion was significantly shorter in the combination therapy group. The mean dosage of misoprostol exhibited a reduction in the combination therapy group compared to the comparison group. Furthermore, the combination therapy group demonstrated a higher rate of complete abortion. Side effects such as abdominal pain and fever were more prevalent among patients in the misoprostol-alone group as opposed to the combination therapy group. The side effect profile exhibited a decrease in the combination therapy group as compared to the comparison group; however, the observed difference did not achieve statistical significance.
Saxena (2021) [16]	India	Women with singleton pregnancy require termination of pregnancy. Without multiple gestations, previous uterine incisions, genital infections, and any underlying medical	Second trimester	*N* = 35 400 mcg of misoprostol plus 40 mg of ISMN intravaginally. A repeat dose of 400 mcg misoprostol plus 20 mg of ISMN was given every 4 h up to a maximum of 5 doses	*N* = 36 400 mcg of misoprostol per vaginum every 4 h up to an upper limit of 5 doses.	Women in the combination therapy group had statistically significantly lower induction abortion time intervals when compared with misoprostol alone. There was no statistical difference in the incidence of adverse effects between the two groups.

ISMN: Isosorbide Mononitrate, IUFD: Intrauterine Fetal Demise

### Description of interventions and comparisons

In three studies [13, 18, 25], women who were going to have an abortion in the first trimester of pregnancy were included in the study. Two studies were conducted on pregnant women at 12–20 weeks of gestation [16, 17], and in the remaining two studies [14, 15], women were included in the study from the 13th to the 24th week of pregnancy. All RCTs had two arms, except the study by Ledingham et al. [18]., which had three arms (a combination of ISMN and misoprostol, ISMN alone, and misoprostol alone). In the combination therapy group, the initial dose of vaginal misoprostol varied from 200 to 800 mcg. The dose of added ISMN also varied from 20 to 60 mg. In some trials, the intervention was done as a single dose, and in other studies, the intervention was repeated with time intervals. More information can be seen in Table 1.

### Risk of bias description

Table 2 shows the risk of biased judgment for included RCTs. Regarding the overall risk of bias, there were three low-risk studies [16, 18, 25], one some-concern study [17], and three high-risk studies [13–15].

**Table 2:** Risk of bias assessment for included randomized controlled trials (RCTs)

First author’s name (year)	Bias arising from the randomization process	Bias due to deviations from intended interventions	Bias due to missing outcome data	Bias in the measurement of the outcome	Bias in the selection of the reported result	The overall risk of bias judgement
Abdel Dayem (2023) [13]	Some concerns	Some concerns	Low	Some concerns	Low	High
Atalla (2022) [14]	Low	High concerns	High concerns	Some concerns	Low	High
Khalifa (2021) [25]	Low	Low	Low	Low	Low	Low
Ledingham (2001) [18]	Low	Low	Low	Low	Low	Low
Mousiolis (2013) [15]	Some concerns	Low	Low	Some concerns	Low	High
Rukkayal Fathima (2016) [17]	Low	Low	Low	Some concerns	Low	Some concerns
Saxena (2021) [16]	Low	Low	Low	Low	Low	Low

In the domain of bias arising from the randomization process, five studies employed a low-risk randomization process and ensured its concealment. In two RCTs, sufficient information about the randomization process and its concealment was not stated [13, 15].

In the domains of bias due to deviations from intended interventions and bias due to missing outcome data, the Atalla et al. (2022) study [14] was rated a high risk of bias because, in some outcomes, the missing outcome data was high and unbalanced, the intention-to-treat was not done, and also no information was included in the field of blinding.

In four trials, the method of measuring the outcome was not deemed inappropriate, but the assessment of the outcome might have been influenced by knowledge of the intervention received [13–15, 17]. All RCTs were rated as low risk of bias in the domain of bias in the selection of the reported result.

### Effect of intervention on the first-trimester abortion

Three studies [13, 18, 25] evaluated the effect of combination intervention compared to vaginal misoprostol alone for the first-trimester abortion. The referenced studies administered vaginal misoprostol at dosages ranging from 200 to 800 mcg and utilized ISMN in quantities ranging from 20 to 40 mg. Khalifa et al. [25], specifically applied a combination therapy consisting of 800 mcg of misoprostol and 40 mg of ISMN as a single dose to the posterior vaginal fornix. We evaluated this regimen against the administration of 800 mcg of misoprostol alone. The results showed that using misoprostol and ISMN together was much more effective than using misoprostol alone to end anembryonic pregnancies, and it was also linked to fewer side effects. Ledingham et al. [18] conducted a three-arm RCT to evaluate the efficacy of 400 mcg of misoprostol combined with 40 mg ISMN administered intravaginally as a single dose, in comparison to two control groups: one receiving 400 mcg of misoprostol intravaginally and the other receiving 40 mg ISMN intravaginally. The study found no significant advantages in combining misoprostol with ISMN over the use of misoprostol alone for pre-operative cervical ripening during the first trimester. Additionally, the incidence of headaches was comparable between the ISMN group and the combination therapy group. Notably, women subjected to the combination therapy experienced side effects consistent with those observed when each agent was administered independently. In a separate trial [13], a single dose of 200 mcg of misoprostol plus 20 mg ISMN placed in the posterior vaginal fornix was compared with 400 mcg of vaginal misoprostol administered four hours before surgical evacuation. While both regimens effectively induced cervical ripening, as evidenced by effacement, dilatation, and softening, the combination therapy proved to be more effective than the misoprostol-only group.

### Effect of intervention on the second-trimester abortion

In four studies, vaginal misoprostol plus ISMN was used to manage second-trimester abortion [14–17]. Two separate clinical trials [16, 17], administered a regimen of 400 mcg of misoprostol and 40 mg of ISMN intravaginally. The subsequent doses consisted of a mixture of 400 mcg of misoprostol and 20 mg of ISMN, administered at four-hour intervals, with a maximum of four to five doses. For comparative analysis, control groups were given 400 mcg of misoprostol intravaginally at the same four-hour intervals, also capped at a maximum of four to five doses.

Atalla et al. [14], administered an initial dose of 400 mcg misoprostol and 20 mg ISMN vaginally. This was followed by 200 mcg of misoprostol at intervals of 4–6 h, up to a maximum of four doses, or until the desired cervical ripening was achieved. The control group administered 200 mcg of misoprostol at intervals of 4–6 h, up to a maximum of four doses, or until they achieved cervical ripening. An additional RCT [15], 200 mcg of misoprostol combined with 60 mg of ISMN was administered vaginally. Six hours after the first administration, they administered a second dose of 60 mg of ISMN if an abortion did not occur. They treated the control group with 400 mcg of misoprostol both vaginally (per vaginum) and orally (per os), followed by a 6-hour interval before administering another 400 mcg of misoprostol vaginally, repeating this process until they achieved fetal expulsion. The meta-analysis findings based on the drug dose are as follows:

### The effect of the intervention on the induction abortion interval

#### Common effect size

**Fig. 2:**
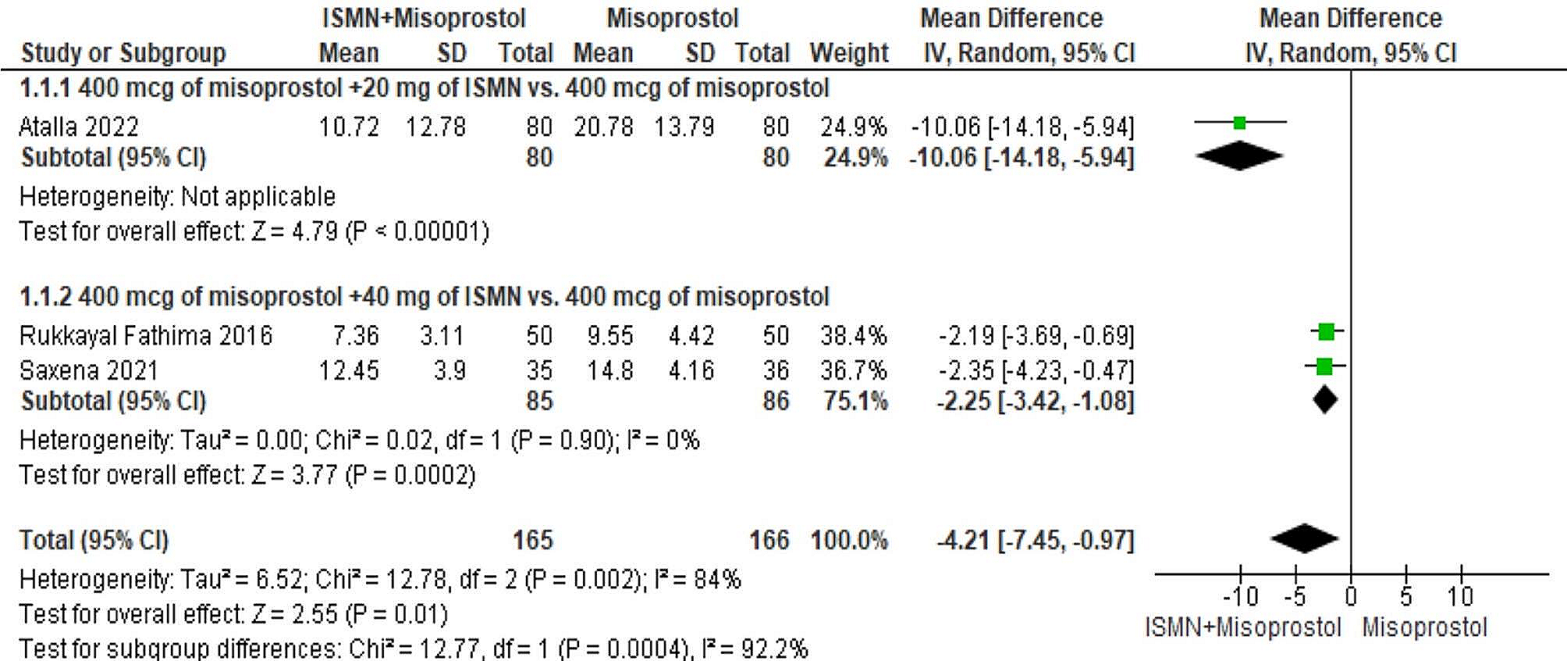
Forest plot of the pooled mean differences (MD) of the effect of the ISMN and misoprostol compared to misoprostol on the induction abortion interval (h) in second trimester abortions

Figure 2 shows the forest plot of the pooled mean differences of the effects of the ISMN and misoprostol compared to misoprostol on the induction abortion interval in the second-trimester abortions. The results of the meta-analysis show that the addition of ISMN to vaginal misoprostol causes a significant shortening of the induction abortion interval for 4.21 h (95% CI: -7.45 to -0.97, *P* = 0.01). Due to the high heterogeneity between studies, a random effect model was used (Chi-squared = 12.87, I-squared = 84%, *P* = 0.002). Due to the limited number of articles, the evaluation of publication bias was not done.

### Subgroup Analysis

Subgroup analysis was carried out based on the dose of ISMN. The findings showed that by adding both doses of 20 and 40 mg of ISMN to 400 mcg of vaginal misoprostol, the induction abortion intervals were significantly shortened.

### The effect of the intervention on the completed abortion rate

**Fig. 3:**
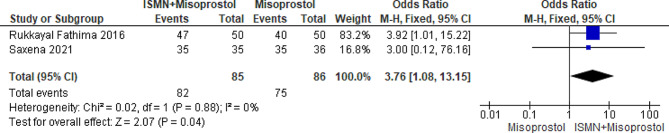
Forest plot of the pooled odds ratio (OR) of the effect of the ISMN and misoprostol compared to misoprostol on the completed abortion rate in second trimester abortions

The effect of the ISMN plus misoprostol compared to misoprostol alone on the completed abortion rate in second-trimester abortions is presented in Fig. 3. The meta-analysis of two RCTs [16, 17] including 171 women showed that the addition of 40 mg vaginal ISMN to 400 mcg vaginal misoprostol compared to 400 mcg vaginal misoprostol alone increased the odds of completed abortion 3.76 times. (95% CI: 1.08 to 13.15, *P* = 0.04). Due to the very low heterogeneity between studies, a fixed effect model was used (Chi-squared = 0.02, I-squared = 0%, *P* = 0.88). Evaluation of publication bias was not done because of the small number of trials.

### Side effects of the intervention

In a review of five studies [14, 16–18, 25], adverse effects associated with the intervention were documented. However, it’s crucial to acknowledge that the primary design of these included RCTs did not aim to evaluate the drug’s safety profile or its spectrum of side effects. Consequently, they lacked the statistical power to identify significant differences in adverse effects. In light of this, our discussion will focus on a qualitative analysis of the side effects observed with combination therapy in comparison to the administration of misoprostol alone.

The most common side effect was abdominal pain [16–18, 26], which favored the misoprostol alone group, ranging from 31.8% [18] to 66.6% [16] in the combination therapy group versus 42.8% [18] to 77.7% [16] in the misoprostol alone group. The most common complication after abdominal pain was headache, which was reported in three studies and was more common in the combination therapy group than in the misoprostol-only group. The combination therapy group reported a headache rate ranging from 36.3% [18] to 38.8% [25], whereas the misoprostol alone group only reported a headache rate in two studies [14, 25], which was lower than the intervention group. The difference in headache between the two study groups was significant only in Khalifa et al.‘s study [25] and was not statistically significant in other studies. Diarrhea and fever were less common side effects in the misoprostol plus ISMN group compared to the misoprostol alone group. Both of these complications in the included RCTs were significantly more common in the comparison group than in the combination therapy group [16, 17, 25].

### Quality of evidence

The GRADE pro-GDT was utilized to assess the quality of evidence for the two main outcomes, and the results are presented in Table 3. The quality of the evidence was moderate for the completed abortion rate, with one downgrade by imprecision. The quality of evidence about the induction abortion interval was very low. It downgraded three levels due to substantial limitations in the risk of bias within the included studies, and serious imprecision and inconsistency.

**Table 3:** GRADE evidence profiles for two main outcomes among the trials included in the meta-analysis

Certainty assessment							№ of patients		Effect		Certainty	Importance
№ of studies	Study design	Risk of bias	Inconsistency	Indirectness	Imprecision	Other considerations	[ISMN+ Misoprostol]	[Misoprostol]	Relative (95% CI)	Absolute (95% CI)		
Induction abortion interval												
3	randomised trials	serious^a^	serious^b^	not serious	serious^c^	none	165	166	-	MD 4.21 lower (7.45 lower to 0.97 lower)	⨁◯◯◯ Very low	CRITICAL
The completed abortion rate												
2	randomised trials	not serious	not serious	not serious	serious^c^	none	82/85 (96.5%)	75/86 (87.2%)	OR 3.76 (1.08 to 13.15)	90 more per 1,000 (from 8 more to 117 more)	⨁⨁⨁◯ Moderate	CRITICAL

CI: confidence interval; MD: mean difference; OR: odds ratio

Explanations

a. Most information is from studies at high risk of bias or some concerns

b. Significant heterogeneity has been found between studies

c. The optimal information size (OIS) criterion is not met

## Discussion

This meta-analysis aimed to determine the efficacy and safety of vaginal ISMN plus misoprostol versus only misoprostol management in first- and second-trimester abortions. This review includes seven randomized trials. Our pooled analysis demonstrated that adding ISMN to vaginal misoprostol significantly shortens the induction abortion interval in the second-trimester abortion.

Based on the presented data, misoprostol is a useful cervical ripening medication that can soften the cervix and increase effacement; however, a synergistic effect occurs when misoprostol and NO donors are combined. NO causes the collagen tissue to produce prostaglandins and other inflammatory mediators, which explains how this process occurs [16].

The meta-analysis of two RCTs [16, 17] including 171 women candidates for the second-trimester abortion showed that the addition of vaginal ISMN to misoprostol compared to vaginal misoprostol alone increased the odds of complete abortion 3.76 times. This clinical finding is critical because many serious problems can happen after an incomplete abortion, including pelvic infections, cervical damage, infertility, severe bleeding or sepsis, death, uterine rupture, uterine perforation, subsequent hysterectomy, failure of multiple organ systems, and/or mental effects [27]. Also, incompleted abortions must be treated surgically, medically, or expectantly [28]; therefore, the consensus is that abortion and post-abortion care place a significant financial burden on society [29]. However, another source of heterogeneity was the variations in the medical assessment of a complete miscarriage. Some studies relied on a history- and examination-based approach, whereas others combined clinical assessment with ultrasound to check for an empty uterus. Also, more research is needed to determine the best combination therapy regimen, including the appropriate dosages and administration schedule because misoprostol and ISMN were administered at different dosages and in different gestational ages.

In some included trials, the rate of side effects, including abdominal pain, fever, and diarrhea, were less in the combination therapy group than in the comparison of only misoprostol. On the other hand, the rate of headache in the combination therapy group was higher than in the misoprostol alone group. There is a pressing need to design robust RCTs that evaluate the safety profile and range of side effects associated with vaginal misoprostol plus ISMN in comparison to misoprostol alone. This is particularly imperative in obstetric management, where safety considerations take on heightened importance due to the dual concern for both the fetus and the mother. According to a meta-analysis conducted in 2023, the incidence of headaches and palpitations was significantly greater in the ISMN group of individuals [26]. Also Abuzaid et al. (2022) confirmed the incidence of maternal headache was significantly elevated in the group receiving both misoprostol and ISMN [30]. Nevertheless, these side effects were deemed clinically insignificant, as none of the women requiring treatment and symptoms were well tolerated. However interpretations must be approached with caution, and multiple aspects concerning women’s safety were carefully examined before the use of ISMN for abortion registration. In common, PGs used for cervical ripening and labor induction are usually administered inpatiently, and fetal monitoring is required. However according to a meta-analysis by Ghosh (2016), the rate of uterine hyperstimulation with FHR alterations with NO donors was lower [31]. If subsequent research validates the safety and efficacy of NO donors, outpatient treatment for abortion may emerge as a viable alternative option. Implementing such a strategy will significantly decrease hospital expenses and potentially enhance maternal contentment with the complete abortion process. However, it is important to conduct a comprehensive cost-effectiveness survey about the utilization of ISMN.

We are conscious that no one method has been confirmed to be effective in determining the degree of certainty associated with the effect estimates that were produced by the meta-analysis. As a result, we used the rigorous procedure that the GRADE Working Group recommended to assess the degree of certainty associated with the evidence. In summary, while there is some evidence to suggest that ISMN plus misoprostol may be beneficial for reducing the induction abortion interval and increasing the completed abortion rate, the certainty of this evidence is low to moderate, indicating that more research is needed to confirm these effects.

The risk of bias among studies contributing to this meta-analysis exhibited variability, with only the three RCTs being appraised as having a low risk of bias. These RCTs were acknowledged as well-conducted and methodologically robust trials. The meta-analysis compared the effects of misoprostol plus ISMN versus misoprostol alone on promoting cervical ripening during labor induction in mothers and babies. It included five RCTs and found that all of them had a low overall risk of bias [30]. In another meta-analysis, 2023 assessed the risk of bias of four studies included that were low [26]. The variance can be attributed to the fact that various authors implement different tools. On the other hand, the process of evaluating the potential for bias is not a precise science and involves a great deal of subjective evaluation. Additionally, it is well known that the researcher’s adopted views and attitudes have an impact on the evaluation process of research studies. Anyway, in our study, it is clear that overall, based on the GRADE assessment, we have very low levels of evidence that ISMN decreases induction abortion interval because the high risk of bias has an unpredictable impact on the results. Therefore, we recommend conducting more RCTs with a more powerful and less biased design and with strong and transparent randomization to strengthen the evidence.

We took several steps to lessen bias, including having two review authors decide whether to include articles, having the two authors independently carry out all data extraction procedures, GRADE evaluations, and risk assessments, and having a third review author resolve arbitration disputes and reevaluate the data as necessary. Second, for us to investigate every potential publication bias, there were not enough studies included in each comparison in the review. Because not all trials recorded data on side effects and their degree of severity, these analyses were frequently limited, or meta-analysis was impossible. This meta-analysis did not conclude that major side effects such as hospital readmissions, mean blood loss volumes, or blood transfusions indicate that such trials should be conducted in the future.

WHO 2022 states that women’s values and choices, intervention acceptability, and resource availability to safely apply the recommended strategy determine implementation [28]. Therefore, to assist women in making treatment decisions, future studies will look at the types of management techniques’ longer-term impacts, such as future fertility rates. Also, future research should, to be precise, help clinicians figure out if the benefits of lessened serousal cervical dilatation are greater than the risks of these drugs’ effects. Patient acceptance, convenience, and satisfaction should also be assessed.

## Conclusion

It seems that the administration of ISMN plus misoprostol through the vaginal route offers potential benefits for women candidates for abortion with fewer uterine cramps compared to prostaglandins alone, which are undesirable before cervical ripening. Given that agents of ISMN and misoprostol are both simple to store, this combined treatment has the potential to be extremely applicable. However, it is necessary to conduct additional trials of high quality to increase the certainty of the evidence about the related outcomes. The results of this study can provide valuable insights for improving counseling and support for non-surgical methods of medication abortion among professionals. Moreover, improves the effectiveness of clinical treatment and reduces the occurrence of unnecessary surgical interventions in the abortion management protocol.

## Data Availability

The datasets used and/or analyzed during the current study are available from the corresponding author upon reasonable request.

## Abbreviations

ISMN: Isosorbide mononitrate
RCTs: randomized controlled trials
RoB2: version 2 of the risk-of-bias
RevMan: Review Manager
